# Delivering Mobile Dentistry to the Geriatric Population—The Future of Dentistry

**DOI:** 10.3390/dj7020062

**Published:** 2019-06-04

**Authors:** Jim Chung

**Affiliations:** Independent Scholar, Don Mills Fairview Dental, 340-5 Fairview Mall Drive, Toronto, ON M2J 2Z1, Canada; jim_chung@sunshine.net

**Keywords:** geriatric dentistry, mobile dentistry, elderly patients, aging, oral health

## Abstract

The human population throughout the world is aging rapidly, and will require the adoption of new modes of dental practice to address the special needs of this demographic. These are some of the reflections of my decade of providing mobile dentistry to geriatric patients.

## 1. Introduction

The mobile practice model being proposed is a significant variation from traditional models that have been pioneered by the military and governmental agencies to service remote populations as early as 1924 [1]. Indeed, the geographic size of Canada has mandated many unique examples of mobile dentistry, such as a train clinic that serviced communities in northern Ontario during the 1960s [2], and recently a semi-retired dentist who sailed the Georgia Strait on the Pacific Coast and provided dental service on board, at the many populated islands where he berthed his sailboat [3].

By projecting population data beginning from 1955, we can determine that over the next twenty years, Canada is expected to double her population of people over the age of 65 to ten million. This will represent less than 20% of Canada’s projected population, and the situation is similar for the US. In Western Europe that population will represent over 25% of its total population, and in Japan that number is approaching 40% of its respective population [4]. This poses a number of fundamental challenges to the traditional model of delivering dentistry.

## 2. The Geriatric Patient Is Not as Mobile

In Canada, people transition to living in a retirement center as they age, because the maintenance of a private residence becomes physically difficult. Seniors generally do not live with their children, and consequently, their children are not available to provide transportation for offsite services such as dentistry. Winter weather further compounds this issue. For the past decade, my dental practice has been providing remote mobile dentistry to a number of local retirement homes on a one-day-per-month frequency. It took considerable lobbying to convince the management of these retirement homes that we (my team comprised of myself and a certified dental assistant) could provide comprehensive, high quality dental care, using completely mobile equipment that we would transport each time. 

The era of complete dentures for all seniors has long past. People are keeping more of their own teeth. Regular hygiene is needed to maintain them and any implants that may also be present. Restorative dentistry and endodontics, rather than extractions and removable prosthetics, have become normalized [5]. This demographic is also historically the most educated and most financially secure, as well as the most dedicated to maintaining oral health [6]. 

Initially my team and I began treating emergencies in hospital and palliative care settings simply because no other dentist was similarly equipped. This consisted of myself and one certified dental assistant working with essentially one operatory. 

Our team also chose to service only local retirement homes in order to occasionally transport patients to our fixed dental clinic for more difficult procedures. In time, management valued us so much that they would feature us in advertising campaigns to recruit new residents.

## 3. They May Have Challenging Physical and Mental Health Issues

Our team chose to target only what are known as “assisted living” retirement centers, where the residents are still physically fit, healthy, and wanting to live an active lifestyle. This is a wealthy demographic conditioned to visiting the dentist regularly. We never solicited patients, only letting the residents know that our team was available every certain Friday of each month. Patients actively seeking our services generally have no health concerns (or stable ones) which would not contraindicate the delivery of elective dentistry.

Patients were required to phone ahead to make a scheduled appointment, although the team did see walk-ins, given the infrequency of our visits. I requested that adult children accompany the patient on the initial visit, especially in cases of early dementia, so that treatment goals and expectations could be determined [7]. Often times, adult children have power of attorney over the finances of their parents. If they refuse to take the time to appear with their parents, in all likelihood they will also refuse to honor any dental fees their parents may generate.

Communication with personal support workers (PSWs) is critical. These are people employed by residents to help them with every aspect of their daily lives. The PSW needs to be educated in how to maintain their client’s oral health and any prosthesis they may have. 

## 4. Dental School Curriculum Needs to Change to Prepare New Graduates for This Population

When I graduated in 1991, there were no specific courses or lectures concerning geriatric dentistry in any Canadian dental school. Fortunately, today about half of all Canadian and American dental schools offer a brief clinical rotation in geriatric dentistry since a didactic course alone is insufficient exposure [8]. Ideally the way forward would be the affiliation of long term care facilities with academic institutions to provide a pool of patients that students could actively treat [6]. After clinical exposure, students reported having multiple false preconceptions that would have prevented them from ever considering a geriatric practice [9]. Brazil was the first country to introduce a speciality in geriatric dentistry in 2001, and New Zealand and Australia have post graduate programs in the field [8].

Both experienced dentists and new graduates need to shift their treatment goals when treating seniors. The goal is not to restore oral function to perfection. The goal is to not complicate their patients’ lives with dental treatment. Instead of treating the patient, care for the patient. Eliminate pain, restore function, treat acute infections and address overt caries. Every retained root does not require extraction, asymptomatic deficient endos can be monitored, and removable prostheses should be offered as an option to implant retained prostheses. Deliver dentistry that will improve the patient, not leave him/her with potential post-op complications [10].

Dry mouth as a result of medication will be a common issue with decay on surfaces that may be hard to access. It may be preferable to restore with amalgam or glass ionomer instead of bonded composites.

Re-educate patients wearing removable prosthesis to remove them as often as possible to facilitate good hygiene, and to wear them as little as possible to improve natural salivary flow.

Seniors often suffer a reduction of motor control/manual dexterity and proper tooth brushing is not possible. Simple procedures like eliminating all loose or open interproximal contacts can do wonders in decreasing the caries rate. Part of the care that we provide is the provision of electric toothbrushes. The ones that are manufactured to run on disposable batteries are reasonable in price. Once patients realize the advantages of powered tooth brushing, they can take the initiative to procure better and more expensive models. 

Often, local anesthetic is not required, so short appointments can still accomplish a significant number of restorative procedures if required.

While the pace of life in the retirement home is slow, and patients often forget their appointments (and your assistant will need to locate them), your visit is often the highlight of their day!

## 5. There Are Few Dental Equipment Manufacturers Providing Mobile Equipment

Our main piece of hardware was the DNTLWorks P2000 [11], a fully-featured delivery system with a full sized air compressor and vacuum pump with a collection jar. This model is now defunct, but preowned units appear on eBay at a very large discount. The unit separates into three modules for easy transportation. Equivalent new models are offered by DNTL Works. Aseptico [12] is another manufacturer of mobile equipment. Both offer a wide variety of models, but the compact ones will not have sufficient suction or air pressure to be useful for a dentist. I would recommend using electric handpieces in conjunction (NSK—Nakanishi Dental Manufacturing Co, Ltd.) since the compressor may not have the capacity to drive handpieces for an extended period of time.

Outdoor garden furniture “zero gravity” lounge chairs are economical, reclining patient chairs when compared to offerings from Aseptico. In the same vein, inexpensive hair salon or spa rolling chairs are perfect for the dentist and assistant to use instead of paying the premium price associated with “real” dental chairs.

All supplies and instruments are carried in a rolling toolbox of the type used by tradesmen like carpenters and plumbers. My assistant was responsible for moving this toolbox while I moved the other items, and we were able to unload the car entirely in just two trips. 

A portable X-ray head is also included in our equipment, and is mounted on a camera monopod (one legged tripod). When used in conjunction with digital sensors instead of film, it allows instant imaging. There are also lightweight handheld mobile X-ray heads available on the market, but at a more premium price [13].

Auxiliary lighting is best accomplished by wearing surgical loupes with on-board lithium- battery-powered LED spotlights.

All equipment is transported in the back of an estate vehicle (station wagon), thereby precluding the need for purchasing a large commercial vehicle.

If you follow a truly mobile dental practice model, even new equipment costs will be small compared to the costs of setting up a traditional fixed dental practice. The most difficult aspect of the model is obtaining cooperation from the operators of the retirement homes. Once this mode of practice becomes mainstream, one could conceivably bring associates on board to service the increased demand of more distant locations. One could perform house calls with a justified surcharge levied for the travel.

## 6. There Is an Oversupply of Dentists in Big Urban Centers

In Canada, there is typically too much competition amongst dentists in the big cities, and a shortage of dentists in the rural regions, especially in the less hospitable northern regions. I have trouble convincing young, new graduates to give up the exciting life of the cities for the isolation of the frontier. Rather than joining a less than busy practice or starting a new practice with zero patients and poor prospects, a new practice model could be a mobile clinic servicing a dozen retirement homes that one could visit twice a month in rotation. Retirement homes are becoming large retirement centers in response to the growing demographic. In the future, progressive centers will include dental clinic infrastructure, and the resident population will be sufficiently large to support a dentist on a permanent, part-time basis. Today, most large retirement homes have a part-time physician on staff. 

In Canada, dental care is not part of the universal health care system as it is in Europe. The perceived high cost of dentistry is the main reason why 100% of the population does not see a dentist regularly. If universal, government-sponsored dentistry becomes a reality in Canada, this model will become more viable, since affordability of dental treatment will no longer be a barrier.

Typically, retirement homes are privately operated, for-profit businesses and charge their residents a premium for their services. The residents will be affluent, they will have been subject to regular dental visits in their youth, and as more of them retain all or most of their teeth, they will seek and require basic dentistry and oral maintenance.

Our reason for beginning this venture was to address a need that patients in our practice with aging parents had broached, and to also counter the often sedentary nature of a conventional clinical practice. We did not approach our mobile dentistry from a business feasibility view, and simply saw patients as the demand dictated. Some days we worked a full day, other days we finished early since we made no attempt to market our services. Since we were careful with our capital investments, had no overhead costs associated with the facilities used, and even attracted new clientele to our home clinic, the venture was always profitable.

The practice of dentistry in the Western world has changed rapidly from decade to decade in the previous century, and will undoubtedly continue to do so at an increased pace. This requires a dentist to be flexible in their definition of a practice, and those who are open to new ideas and new trends will be the ones with the most successful and satisfying practices in the 21^st^ century. Studies of traditional mobile dental units show they are a temporary, stop-gap measure to address inequalities in service to targeted populations, and often are dependent on philanthropic or transient government funding. The expectation is that the population awareness for dental treatment will rise, attracting the building of permanent dental facilities by private clinicians [14,15]. There have been studies that have mathematically modeled this form of mobile dentistry to determine which parameters can affect its business viability [16]. Fortunately, the model discussed here suffers from none of those intrinsic disadvantages. Instead of a poor non-insured patient base, we deal with an affluent subset—many with private dental insurance. Instead of unknowledgeable patients, we have a motivated population with a deeply ingrained desire to seek out services. Instead of a large service area of low population density requiring considerable travel, our travel is limited, and one large residential retirement complex yields hundreds of potential patients. Within the scenario proposed in this paper, the ultimate building of permanent dental clinics within a larger medical health center in large retirement homes could be accomplished by the home operator themselves, or in partnership with health professionals. Until this becomes a reality in the near future, the portable dental practice model is very feasible for today’s general practitioner looking for a change, to get a business advantage over competing dentists, to experience interaction with seniors, or all of the preceding.
